# A longitudinal study of naloxone opioid overdose awareness and reversal training for first-year medical students: specific elements require reinforcement

**DOI:** 10.1186/s12954-022-00656-y

**Published:** 2022-07-02

**Authors:** Reena K. Sandhu, Michael V. Heller, Jack Buckanavage, Benjamin Haslund-Gourley, Joshua Leckron, Brady Kupersmith, Nathaniel C. Goss, Kyle Samson, Annette B. Gadegbeku

**Affiliations:** 1 Drexel University College of Medicine, Philadelphia, PA USA; 2 Department of Family, Community and Preventive Medicine, Drexel University College of Medicine, Philadelphia, PA USA

## Abstract

**Background:**

The opioid epidemic is a progressively worsening public health crisis that continues to impact healthcare system strategies such as overdose reversal and destigmatization. Even among healthcare professionals, there remains a lack of confidence in naloxone administration and a prevalence of stigma. While training can play a major impact in reducing these shortcomings, the long-term effectiveness has yet to be characterized in training healthcare professionals. This study examined the long-term retention of opioid overdose awareness and reversal training (OOART) by evaluating performance at two-time intervals, immediately post-training and at a 3-month follow-up.

**Methods:**

Voluntary training was offered to first-year (M1) medical students at the Drexel University College of Medicine in 2021. At this training, 118 students completed training, 95 completed the post-training survey, and 42 completed the 3-month follow-up.

**Results:**

Opioid reversal knowledge questions assessed significantly increased scores post-training and at the 3-month follow-up. In three of the attitude questions, scores were improved at both follow-up timepoints. In addition, three attitude questions indicating a participant’s confidence to respond to an opioid overdose situation increased directly after the training, but regressed at the 3-month follow-up. The remaining questions did not show any statistical difference across the survey intervals.

**Conclusions:**

This study establishes that while OOART provides participants with the knowledge of how to respond to an opioid overdose, the retention of this knowledge at a 3-month interval is reduced. The results were mixed for longitudinal assessment of participant’s attitudes toward people with opioid use disorder. Some positive increases in attitudes were retained at the 3-month interval, while others trended back toward pre-training levels. These results support the effectiveness of the training but also provide evidence that OOART must be reinforced often.

**Supplementary Information:**

The online version contains supplementary material available at 10.1186/s12954-022-00656-y.

## Introduction

Opioid-related overdose deaths have been notably rising for the past two decades [1–3]. Since 1999, nearly 841,000 individuals in the US have died from a drug overdose, of which over 500,000 were opioid-related deaths [4]. As the supply shifted to include a growing proportion of synthetic opioids of higher potency, such as heroin and fentanyl, there has been a concomitant spike in opioid-related deaths [5–7]. Synthetic opioids have significantly contributed to this escalation, evidenced by a 1040% increase in synthetic opioid-involved deaths in the US from 2013 to 2019 [8]. When considering Philadelphia, a city particularly affected by the opioid overdose epidemic, unintentional drug overdose deaths have increased by 6% from 2019 to 2020, with 86% of cases involving opioids [9]. Overlaid upon this progressive public health crisis is the COVID-19 pandemic [10–13]. Recent data suggest that total opioid overdose deaths in the US have increased 28.5% when comparing the statistics from April 2019–2020 (56,064) to April 2020–2021 (75,653) [14]. These sobering statistics expose the vast amount of work necessary to address the ongoing opioid overdose epidemic and underscore the urgent demand for improvement in access, resources, and care available to individuals struggling with opioid use disorder (OUD) [15–17].

One method of addressing the opioid overdose crisis is improved access to naloxone, a lifesaving opioid overdose reversal medication, in health care settings and communities with high rates of opioid overdose [18–20]. Naloxone standing orders allow anybody to obtain naloxone from a pharmacy without a prescription from a physician, and their implementation has had a demonstrable positive impact [21–23]. Xu and colleagues noted dramatic increases in total naloxone orders dispensed when standing orders were implemented in states, jumping from 1488 in 2007 to 147,457 in 2016 [24]. More recently, there have been expansions of naloxone access laws in some states, which included adopting co-prescribing requirements of naloxone when physicians prescribe opioids [21, 25, 26].

Despite the positive impact standing orders have had in easing access to naloxone, it is apparent that increasing naloxone access will not be sufficient in ending this epidemic [27]. Another intervention to maximize and promote the benefits of naloxone is by increasing overdose reversal knowledge among healthcare providers [28, 29]. Physician-held biases can manifest in care of individuals with OUD through underutilization of buprenorphine prescription capacity, avoidance of association with individuals with OUD, or provision of suboptimal medical care—all of which can lead to worsened physical and psychosocial health outcomes and future health-seeking behavior [30]. To address this, training of internal medicine physician residents has demonstrated that opioid overdose awareness and reversal training (OOART) can lead to increased naloxone prescription rate as well as positive improvements in self-reported attitudes and willingness to provide harm-reduction centered care [31].

Thus, it is evident that intervening earlier and providing OOART to future healthcare providers during their education can help preemptively address gaps in knowledge or biases prior to beginning clinical practice [32]. Findings by Bascou et al. [33] suggest statistically significant improvements in first-year medical students’ subjective attitude and objective knowledge measures following OOART. The results from Goss et al. [34] reinforce these findings, and additionally show equivalent efficacy in improvement when comparing in-person training versus online training of medical students, necessitated by the COVID-19 pandemic. Other pre-professional school programs with similar OOART paradigms have experimented with embedding training into school curricula to reach all students, and their results echo similar improvements in knowledge, attitudes, and preparedness [35–39].

OOART has been shown to powerfully address subjective stigma, misconceptions, and gaps in opioid overdose knowledge. However, it is crucially important to monitor the longevity of these gained improvements following OOART, not only as a metric of the strength of the OOART but also as a surrogate for expected changes in clinical behavior. To this end, Jacobson et al. [40], examined the retention of overdose recognition and naloxone reversal knowledge at six months following training in first- and second-year pharmacy students. They found maintenance in knowledge regarding naloxone pharmacology and response to opioid overdose [40]. Research in law enforcement officers who received naloxone training demonstrates retention of overdose recognition and reversal knowledge at the six-month follow-up [41]. The value of OOART for medical students was evident in a study that compared knowledge retention at six months in students who have received OOART to untrained students [42]. Of note, both groups in this study had a significant increase in knowledge and attitudes and the analysis grouped questions rather than analyzing each separately, both of which are significant limitations on the impact of the findings presented.

Drexel University College of Medicine’s student-led Narcan Outreach Project (NOP) provides a comprehensive OOART and take-home naloxone to future healthcare provider students and the general public. NOP delivered online OOART to first-year medical students in Fall 2021 and collected pre-, post-, and 3-month post-training survey results. The focus was to expand upon previous findings and rigorously examine the longevity of both knowledge and attitude metrics in our cohort. We also looked to identify which (if any) specific components indicated training decay to better tailor current OOART and promote comprehensive and long-lasting change in participants’ awareness and knowledge for OUD and naloxone administration. The objectives of this paper were to (1) determine the efficacy of our OOART and (2) compare knowledge and attitude metrics prior to, immediately after, and three months after receiving OOART.

## Methods

### Development of OOART

The OOART was developed by a group of medical students at Drexel University School of Medicine with assistance from faculty advisors and members of the Philadelphia Department of Public Health, as described in Goss et al. [10]. In brief, several individuals involved in the implementation and design of the study have lived-experience with OUD. In addition to providing practical knowledge of naloxone administration, our OOART provides unique insights into the sociohistorical development of the opioid epidemic and biopsychosocial considerations pertinent to those suffering from OUD. The OOART is comprised of a PowerPoint presentation, which was split into seven sections: (1) Opioid Basics, (2) Introduction to the Opioid Epidemic, (3) A Brief History and the Aftermath, (4) The Experience of OUD, (5) Race/Ethnicity Disparities in the Opioid Epidemic, (6) OUD Treatment and Harm Reduction as a Tool, and (7) Overdose Reversal, Naloxone Administration, and Post-Reversal Care. A breakdown of the presentation has been described previously [10], with a detailed outline of the discussion and assessment topics depicted in Additional file 1: Table S1. Embedded within Sect. 7, Overdose Reversal and Naloxone Administration, were four videos produced by NOP simulating an overdose, in which one student acted as an individual who overdosed and the other as a ‘Good Samaritan’ performing the overdose reversal.

Readers should note that although the nasal form of naloxone, Narcan, was used and distributed in these training sessions, for consistency, we will refer to the opioid antagonist only as its generic name throughout the remainder of the paper.

### Survey

Surveys were administered prior to training (pre-training), directly after training (post-training) and after 3 months (3-month-post-training). The pre-survey collected demographic information, including age, gender, and employment status. It also questioned participants if they have previously trained in the use of naloxone, if they have witnessed an overdose, if they have administered naloxone, and whether or not they are currently carrying naloxone. The remaining questions were identical between the pre-, post-, and 3-month-post-training surveys and were used to obtain data on knowledge and attitudes.

In terms of attitudes, the survey included 12 questions. These questions were further divided into three subsections: (a) “Attitudes Towards Naloxone and Overdose Reversal,” (b) “Attitudes Towards Individuals with OUD”, and (c) “Self-Confidence in Using Naloxone and Handling an Overdose.” Our modified survey included six questions pertaining to “Attitudes Towards Naloxone and Overdose Reversal” or “Self-Confidence in Using Naloxone and Handling an Overdose.” We also devised five additional questions to address “attitudes towards individuals with OUD.” The exact wording and categorization of the questions are presented in Additional file 1: Tables S2 and S3. All attitudes questions were scored on a 5-point Likert Scale (Completely Disagree = 1; Disagree = 2; Unsure = 3; Agree = 4; Completely Agree = 5). To assess knowledge, the survey included 3 multiple-choice fact-based questions adapted and shortened from the Opioid Overdose Knowledge Scale [10,34]. The competency questions were scored as “1” for correct or “0” for incorrect. This allowed for comparison between individual questions and between overall percent correct.

### Delivery of OOART

The trainings were conducted online in Philadelphia between October 2021 and January 2022. Participants were first year medical students. Pre-surveys were distributed via email containing a Google survey link prior to the online session. Participants were instructed to complete the survey prior to the presentation. At the end of the session, participants completed the post-survey using a link provided in a follow-up email. Three months after the online OOART, the 3-month-post-training survey was distributed to all participants via email. As with many other opioid overdose prevention programs, naloxone was distributed to all participants who completed the pre- and post-training surveys.

### Data analysis

Analysis for statistical differences between surveys was conducted using a nonparametric One-Way ANOVA, with post hoc Tukey's multiple comparisons test used to examine the three timepoints for statistically significant differences. Calculated *p* values of < 0.05 were considered statistically significant. Descriptive statistics were also used to display the pre-, post-, and 3-month-post- mean and standard deviation for each question. Figures are generated using GraphPad Prism Software Version 8.

### Recruitment and inclusion/exclusion criteria

Medical students were recruited predominantly through emails and by word of mouth. All participation was voluntary. There were no specific inclusion criteria. All demographic data were included in the results, regardless of whether the remainder of the survey was complete or incomplete. Responses to individual questions were excluded from the analysis when participants answered the pre-training question but failed to respond to the corresponding post-training question or vice versa.

## Results

Participants were composed of first-year medical students with an average age of 24 years old and an age range of 21 to 36 years. The self-reported gender was skewed toward female with 36% males, 63% female, and 0% prefer not to say/non-binary. Over 96% of participants identified their employment status as ‘student’, while 2.5% were Full-Time employees and 0.85% were Part-Time. While 14% of participants reported they had previously attended a naloxone training, only 0.84% reported regularly carrying naloxone on their person. The respondents to the pre-survey totaled 118 while the post-survey responses totaled 95 for an attrition rate of 20% loss in respondents. The 3-month survey respondents totaled 42, for an attrition rate of 56% from post-survey responses.

**Fig. 1:**
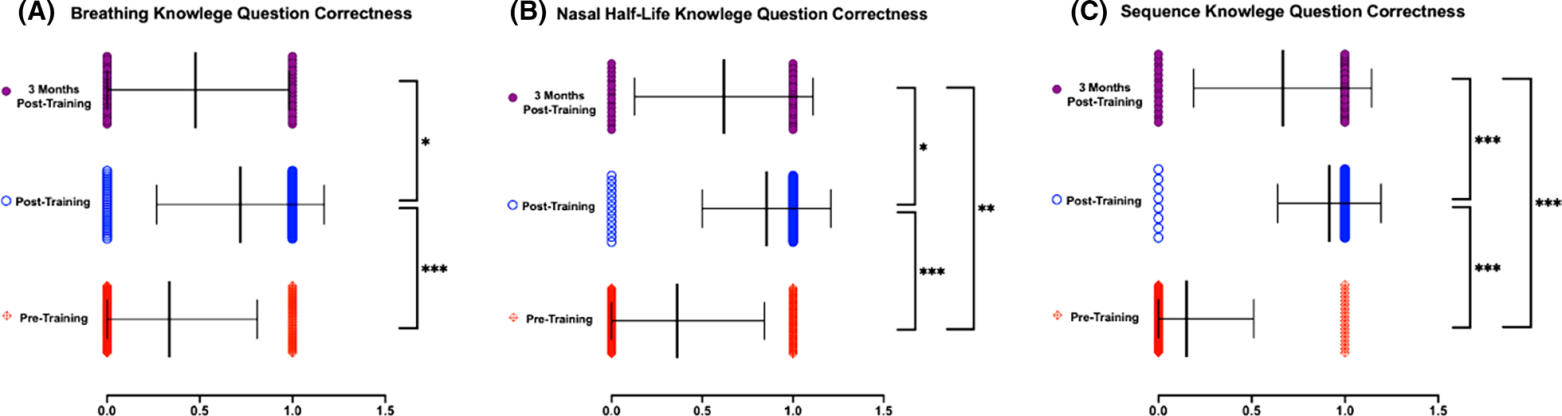
Knowledge questions assessed before, directly after, and 3-months after reviewing OOART training. Data presented as Mean ± SEM. **A** Breathing **B** Nasal half-life **C** Sequence knowledge. *n* = 118 for pre-training, *n* = 95 for post-training, *n* = 42 for 3-month post-training survey respondents. *p < 0.05, **p < 0.01, ***p < 0.001

**Fig. 2:**
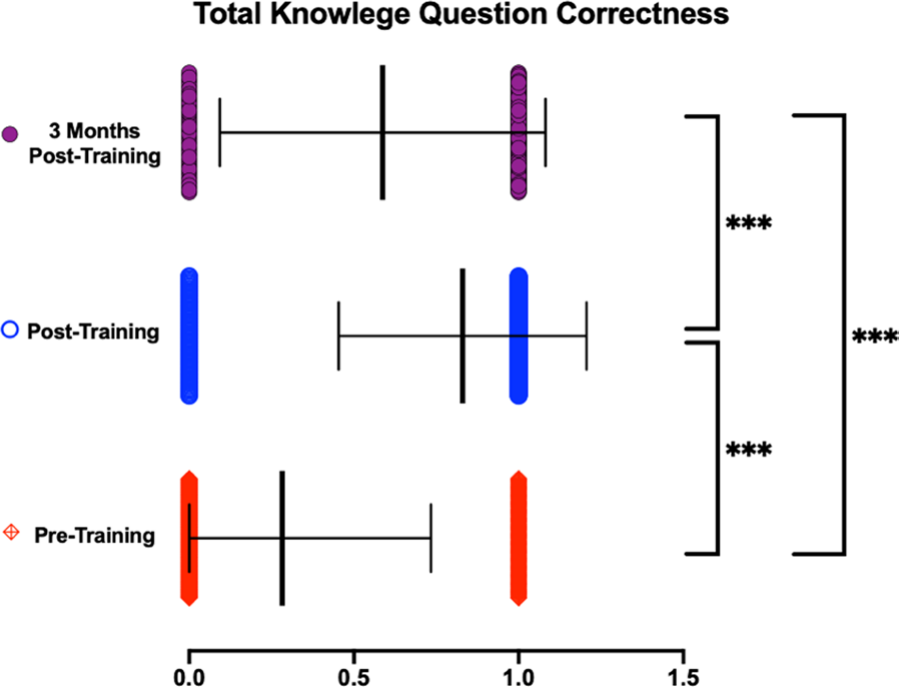
Average of All 3 knowledge questions assessed before, directly after, and 3-months after reviewing OOART training. Data presented as Mean ± SEM. *n* = 118 for pre-training, *n* = 95 for post-training, *n* = 42 for 3-month post-training survey respondents. ***p < 0.001

Figure 1 shows improvement in accuracy between pre- and post-training survey responses in all three knowledge questions. The percent increase in correct answers was 14%, 26%, and 52% between pre- and 3-months-post-training for Questions 1, 2, and 3, respectively. Comparisons between timepoints revealed significant decreases in knowledge at the 3 months post-training timepoint—a 24%, 24%, and 25% decrease between post- and 3-month-post-training in Questions 1, 2, and 3, respectively. Figure 2, which shows the average combined percent correct for all three knowledge questions, shows an overall 54.7% increase in correct answers immediately following training and a 24% decrease in correct answers between post- and 3-months-post-training, resulting in an overall 30.44% increase from participants’ baseline to 3-months post-training.

**Fig. 3:**
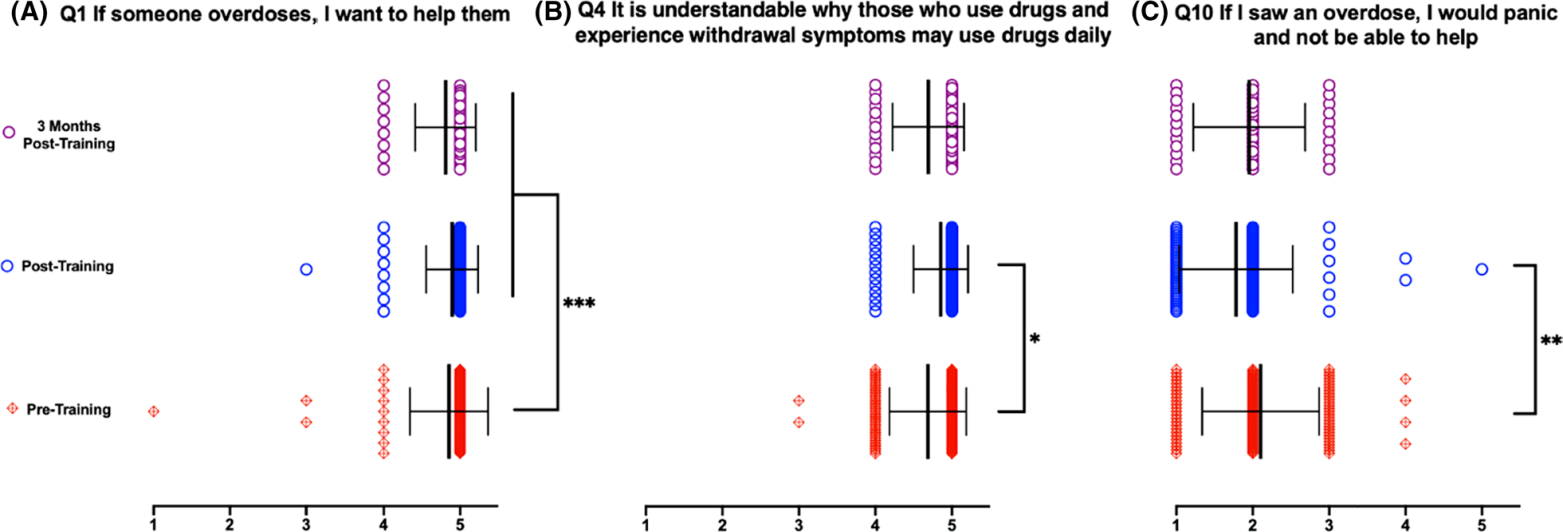
Attitude questions assessed before, directly after, and 3-months after reviewing OOART training with retention of training objectives. Data presented as Mean ± SEM. **A** help **B** understanding **C** panic response. *n* = 118 for pre-training, *n* = 95 for post-training, *n* = 42 for 3-month-post-training. *p < 0.05, **p < 0.01, and ***p < 0.001. 1 = Strongly disagree, 2 = Disagree, 3 = Indifferent, 4 = Agree, 5 = Strongly Agree

Additional file 1: Figure S1 presents all twelve attitude and awareness question responses with appropriate statistical significance. Figure 3 presents the three question responses (Questions 1, 4, and 10) wherein there were significant improvements in attitude between pre- and post-training that were maintained at the 3-month timepoint. Question 1 aims to determine the level of comfort to assist a person experiencing an opioid overdose. At both post- and 3-month-post-training, participants rated their desire to help during an opioid overdose significantly higher than at the pre-training survey level. Question 4 interrogates the participant’s understanding of the struggles someone with opioid use disorder faces. While significant increases in understanding were measured between pre- and post-training responses, there was no significant difference between post- and 3-month-post-training, supporting a maintenance of participants’ attitudes. Lastly, Question 10 determined if the participant feels they would be unable to respond to an opioid overdose situation and panic instead. Participants responded with a significant decrease in their presumed tendency to panic post-training, and that decrease was maintained during the 3 month post-training response, indicating no significant difference between post- and 3-month-post-training.

**Fig. 4:**
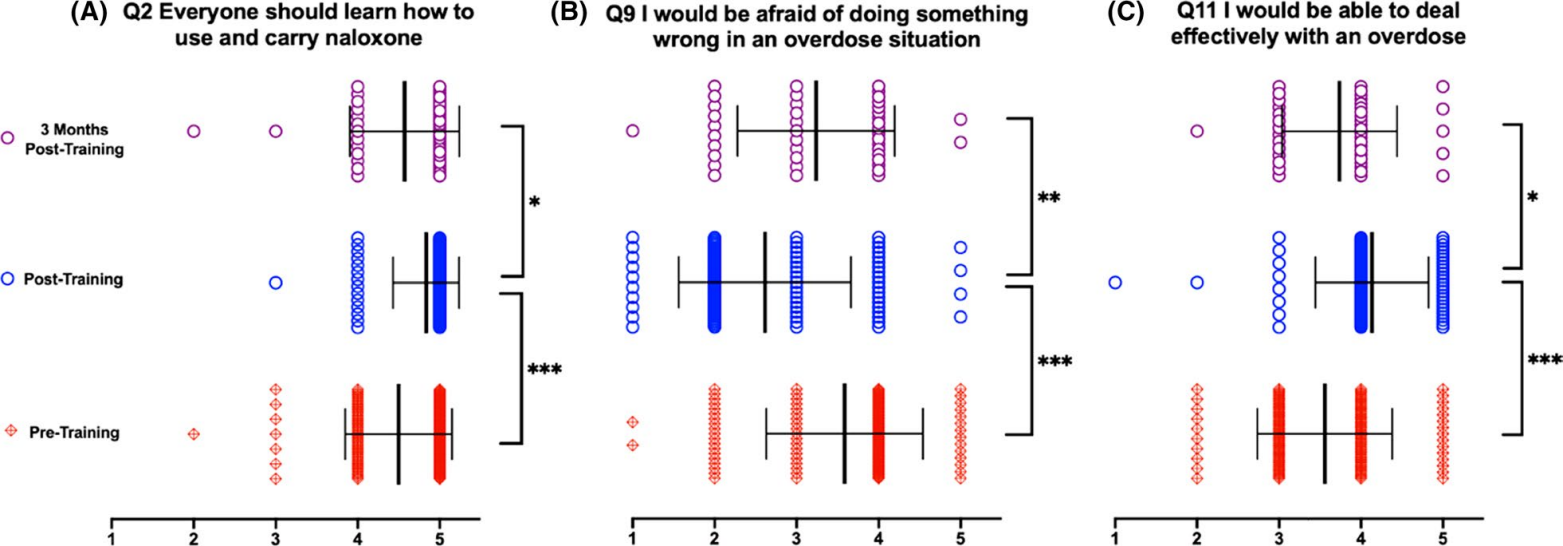
Attitude questions assessed before, directly after, and 3 months after reviewing OOART training with regression to pre-training levels. **A** Learning opinion **B** Fear response **C** Effective response. *n* = 118 for pre-training, *n* = 95 for post-training, *n* = 42 for 3 month post-training. *p < 0.05, **p < 0.01, and ***p < 0.001. 1 = Strongly disagree, 2 = Disagree, 3 = Indifferent, 4 = Agree, 5 = Strongly Agree

Three of the twelve attitude questions (Questions 2, 9, 11) exhibited regression at 3 months post-training from the progress gained after training back to the baseline pre-training averages, ultimately making all comparisons between pre- and 3-month-post-training results nonsignificant (Fig. 4). Question 2 asked whether everyone should be trained to use and carry naloxone. There was a significant increase in participants' belief directly after the training; however, comparison between the post- and 3-month-post-training results show a significant decrease in that belief. Question 9 queried the participants' fears of doing something wrong when responding to an overdose situation. While participants reported a significant decrease in their fear of doing something wrong directly after the training, their fear level significantly increased from post-training results at the 3-month post-training survey. Lastly, Question 11 polled participants in their ability to effectively deal with an overdose. There was a significant increase in the post-training response reflecting improved ability to respond to an overdose, yet their self-reported ability significantly decreased between post- and 3-months-post-training.

## Discussion

Despite increasing awareness of naloxone, the opioid epidemic remains a prominent public health issue. This reality makes OOART for future healthcare providers more crucial than ever, as properly responding to opioid overdoses and reducing stigma can play a meaningful role in mitigating this crisis. As demonstrated by Bascou et al. [9] and Goss et al. [10], OOART delivered to medical students can lead to significant positive improvements in knowledge and attitudes, while reducing stigma immediately following training. Building upon this existing literature and methodology, our longitudinal analysis of acquired knowledge and attitude improvement 3 months following OOART indicate that depreciation in both subjective and objective measures occurs, but does not fully regress to the pre-training baseline. When examining the three Knowledge questions in aggregate as depicted in Fig. 2, comparisons between the 3 timepoints illustrate this trend of an overall sizable improvement and retention in knowledge after 3 months, which is consistent with the literature and findings previously discussed [40–42]. The questions pertaining to the half-life of naloxone and the sequence of steps in an overdose demonstrated statistically significant differences in pre-training and 3-months-post-training, whereas the remaining knowledge question, which addressed rescue breathing knowledge, did not. This finding indicates that not all knowledge was retained equally in participants, representing a content area that can be emphasized differently in future OOART.

When examining the 12 Attitude questions, the results do not all follow the aforementioned pattern of improvement and retention in attitude and biases after 3 months. Attitude Questions 1, 4, and 10 (which assess willingness to help, understanding why people use substances, and likelihood to panic if witnessing an overdose, respectively) showed statistically significant positive improvement and retention in beliefs compared to pre-training data. Question 1, in particular, assesses participants on what ultimately is one of DUCOM NOP’s guiding principles: knowledgeable and unbiased bystander intervention in the event of an overdose. Prior to receiving the OOART, participants varied widely in their responses, with some selecting that they would not want to help. Following the training and 3 months afterwards, every single participant stated that they agree or strongly agree with helping in this situation. This finding powerfully underscores the lasting value of this OOART as well as the potential clinical relevancy and behavior from this change in attitude.

Attitude Questions 2, 9, and 11 (which assess opinions about whether the public should have knowledge of use and carry of naloxone, fear of making a mistake in an overdose, and self-confidence in ability to respond to an overdose, respectively) interestingly reflect a regression of improvement in attitude and confidence 3-month-post-training back to near pre-training levels. When considered in the context of the depreciation in objective knowledge, the findings from Questions 9 and 11 can be interpreted as a reflection of the participants’ self-awareness of their loss in factual knowledge, leading to lower reported levels of self-efficacy. Therefore, developing strategies to enhance factual knowledge retention may help mitigate the losses in self-preparedness in an overdose. Lastly, the remaining Attitude Questions 3, 5, 6, 7, 8, and 12 did not show statistically significant changes when compared across the three timepoints. These questions demonstrated high favorable attitudes prior to even receiving the OOART, reflective of the participants’ high baseline attitudes and opinions as future physicians. The results from our study extend the preliminary findings of Moses et al. [42] by providing further granularity upon which specific attitudes and beliefs diminished over time.

This paper represents the first quantitative analysis of individual differences in responses to both subjective and objective measures across a longitudinal study after participants received OOART. This longitudinal OOART post-training reassessment will continue to serve as the foundation of the DUCOM Naloxone Outreach Program’s educational outreach, thus allowing for teams to continuously improve the content, delivery, assessment, and impact of the training. Future trainings can iteratively analyze the content and structure of knowledge and attitude questions that showed marked decline over three months, allowing for a precise understanding of what information may need to be more strongly reinforced, presented differently, or assessed in a differently phrased manner. In addition, trainings based on findings from memory and recall studies can explore spaced-repetition or other longitudinal-based learning approaches, and examine whether these strategies enhance knowledge recall and attitude improvement retention. These approaches can include providing participants with a brief video re-emphasizing the key points from the OOART along with formative self-assessments to enhance the encoding, storage, and ultimately recall of this vitally important knowledge.

This study’s most significant limitation includes the attrition rate of responses at the 3-month follow-up, wherein only 44% of participants who completed both the pre- and post-OOART survey completed the 3-month post-training survey. Participants who were more likely to be motivated and interested in completing a 3-month post-training survey may not represent the sample of participants studied. Of note, our attrition rate is comparable to those disclosed by Nath et al. [41] and Moses et al. [42]. Finally, these findings are limited in their generalizability to non-medical student populations, as our sample solely consisted of first-year medical students in Philadelphia.

## Conclusion

With the increasingly devastating effects of the opioid epidemic, especially in the setting of COVID-19, physicians have a key role in combating this crisis. Previous studies by the Narcan Outreach Project at the Drexel University College of Medicine, as well as other healthcare provider education programs, have demonstrated that OOART increases knowledge and reduces stigma in students toward the use of opioids. Our study expanded these results and evaluated whether changes were consistent across a 3-month time-span. While these initial improvements remained at both post-training and a 3-month interval, we observed a reduction in the training effect at 3 months versus directly after the training. These results provide future direction for potential strategies of continual opioid education and refresher courses to maintain the improvements achieved directly after the OOART, with the hope of seeing improved future clinical behavior with and outcomes for individuals experiencing OUD.

## Supplementary Information

**Additional file 1: Figure S1** All Attitude questions. **Table S1** Outline of online trianing components and content. **Table S2**. Attitude questions asked in Pre-, Post-, and 3mo. Post-survey. **Table S3**. Knowledge questions asked in Pre-, Post-, and 3mo. post-survey

## Data Availability

The datasets used and/or analyzed during the current study are available from the corresponding author on reasonable request.
