# Supplementary material for: A longitudinal study of naloxone opioid overdose awareness and reversal training for first-year medical students: specific elements require reinforcement
Source: Harm Reduct J. 2022 Jul 2;19:70. doi: 10.1186/s12954-022-00656-y (PMC9250225; doi:10.1186/s12954-022-00656-y)
Supplement: Supplementary file 1 — Additional file 1: Figure S1 All Attitude questions. Table S1 Outline of online trianing components and content. Table S2. Attitude questions asked in Pre-, Post-, and 3mo. Post-survey. Table S3. Knowledge questions asked in Pre-, Post-, and 3mo. post-survey [file 12954_2022_656_MOESM1_ESM.docx]

**Title Page**

**Title:** A Longitudinal Study of Naloxone Opioid Overdose Awareness and Reversal Training for First-Year Medical Students: Specific Elements Require Reinforcement

**Authors:** Reena K. Sandhu^1^, Michael V. Heller^1^, Jack Buckanavage^1^, Benjamin Haslund-Gourley^1^, Joshua Leckron^1^, Brady Kupersmith^1^, Nathaniel C. Goss^1^, Kyle Samson^1^, and Annette B. Gadegbeku^2^

**Institutional Affiliation:**

^1^Drexel University College of Medicine, Philadelphia, Pennsylvania, USA. ^2^Department of Family, Community & Preventive Medicine, Drexel University College of Medicine, Philadelphia, Pennsylvania, USA.

**Corresponding Author:** Reena K. Sandhu, [rks75@drexel.edu](mailto:rks75@drexel.edu)

**Supplemental Figure 1**


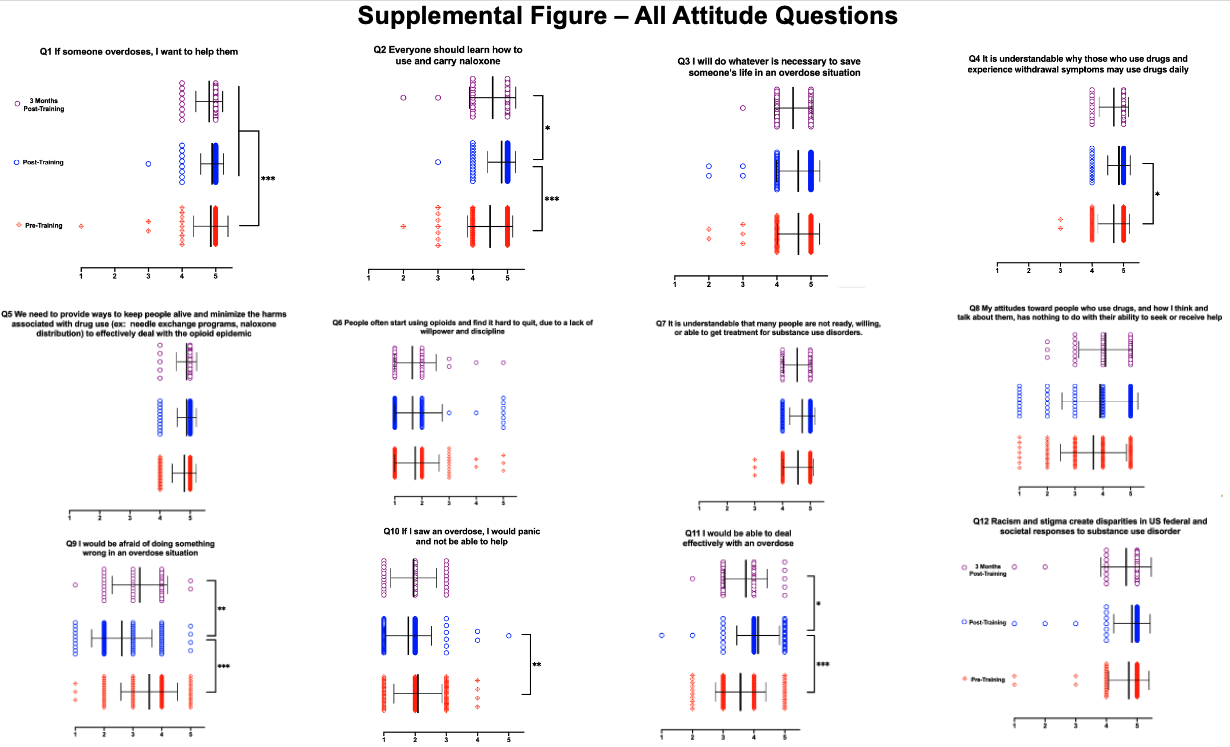


**Supplemental Table 1**

**
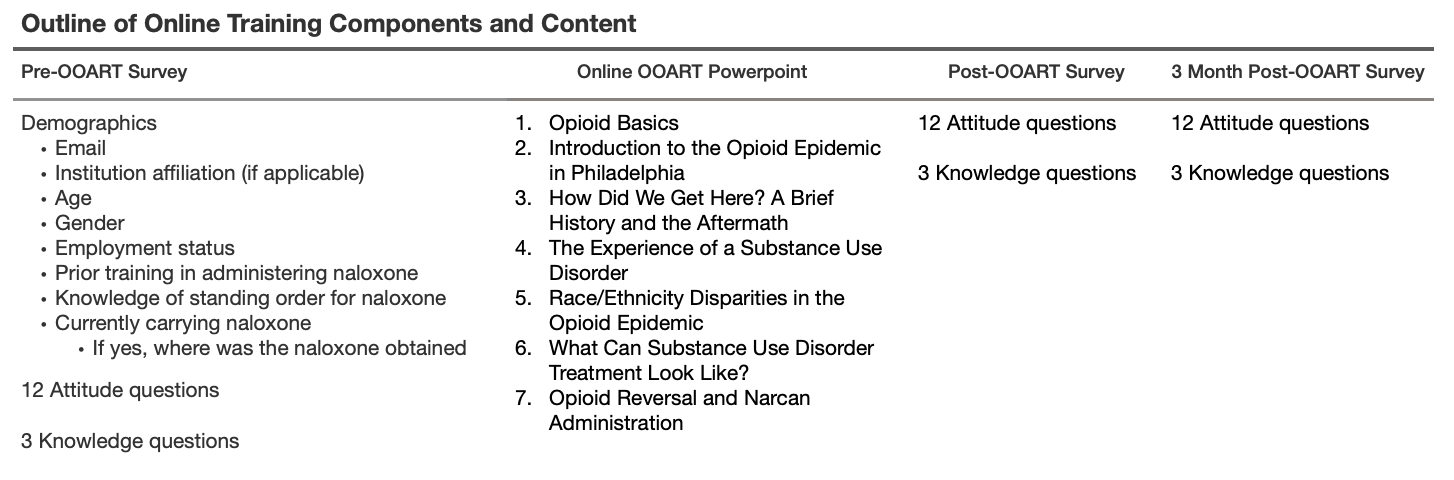
**

**Supplemental Table 2**


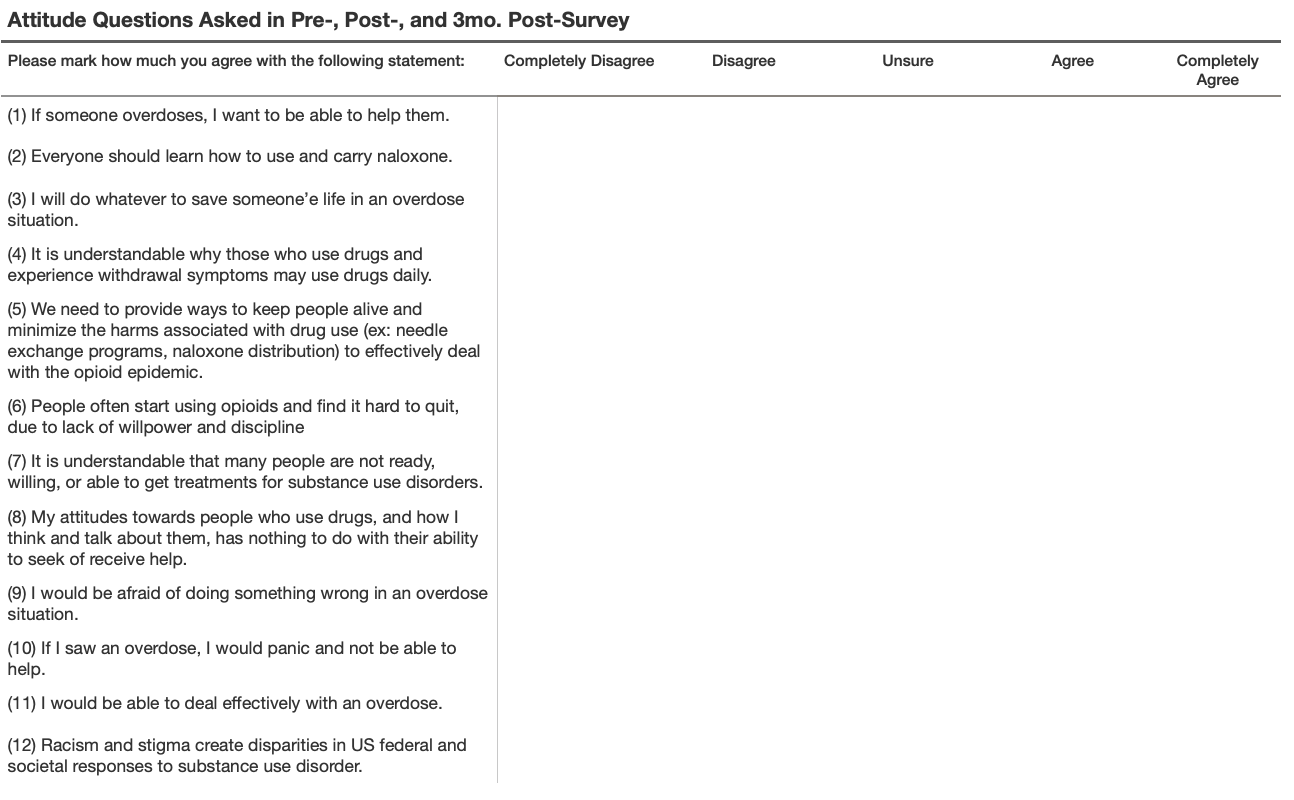


**Supplemental Table 3
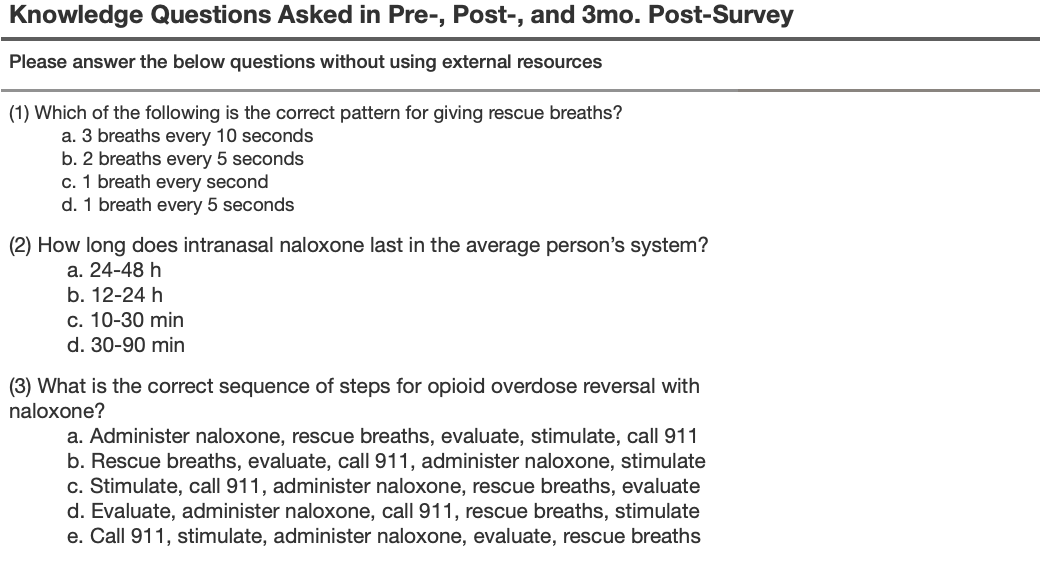
**
